# Epigenetic reader BRD4 inhibition as a therapeutic strategy to suppress E2F2-cell cycle regulation circuit in liver cancer

**DOI:** 10.18632/oncotarget.8701

**Published:** 2016-04-12

**Authors:** Seong Hwi Hong, Jung Woo Eun, Sung Kyung Choi, Qingyu Shen, Wahn Soo Choi, Jeung-Whan Han, Suk Woo Nam, Jueng Soo You

**Affiliations:** ^1^ Konkuk University Medical Center, School of Medicine, Konkuk University, Seoul 143-701, Korea; ^2^ Functional RNomics Research Center, College of Medicine, The Catholic University, Seoul 137-701, Korea; ^3^ Research Center for Epigenome Regulation, School of Pharmacy, Sungkyunkwan University, Suwon 440-746, Korea

**Keywords:** BET protein, epigenetic components, JQ1, hepatocellular carcinoma (HCC), E2F

## Abstract

Deregulation of the epigenome component affects multiple pathways in the cancer phenotype since the epigenome acts at the pinnacle of the hierarchy of gene expression. Pioneering work over the past decades has highlighted that targeting enzymes or proteins involved in the epigenetic regulation is a valuable approach to cancer therapy. Very recent results demonstrated that inhibiting the epigenetic reader BRD4 has notable efficacy in diverse cancer types. We investigated the potential of BRD4 as a therapeutic target in liver malignancy. BRD4 was overexpressed in three different large cohort of hepatocellular carcinoma (HCC) patients as well as in liver cancer cell lines. BRD4 inhibition by JQ1 induced anti-tumorigenic effects including cell cycle arrest, cellular senescence, reduced wound healing capacity and soft agar colony formation in liver cancer cell lines. Notably, BRD4 inhibition caused MYC-independent large-scale gene expression changes in liver cancer cells. Serial gene expression analyses with SK-Hep1 liver cancer cells treated with JQ1 to delineate the key player of BRD4 inhibition identified E2F2 as the first line of downstream direct target of BRD4. Further experiments including chromatin immunoprecipitation (ChIP) assay and loss of function study confirmed E2F2 as key player of BRD4 inhibition. Overexpressed E2F2 is a crucial center of cell cycle regulation and high expression of E2F2 is significantly associated with poor prognosis of HCC patients. Our findings reveal BRD4-E2F2-cell cycle regulation as a novel molecular circuit in liver cancer and provide a therapeutic strategy and innovative insights for liver cancer therapies.

## INTRODUCTION

The function of the genome is determined by the epigenome, which consists of a record of the chemical changes to DNA and histone, and more broadly the compactness of the chromatin structure [1, 2]. Chromatin structure which is a major determinant for gene expression potential is signed by the role of epigenome component [3]. Epigenome component can be divided into three proteins; writers, erasers and readers [4]. Three proteins’ corporation is crucial for the dynamic process of epigenome regulation. Traditionally cancer has been considered as a disease driven by genetic anomalies, but it is now clear from extensive proteomic and genomic studies that misregulation of epigenetic components also play a significant role in oncogenesis [4, 5]. There has been tremendous effort to develop small molecules which can target dysregulated epigenetic components. A number of epigenetic inhibitors have been developed; targeting DNA methyltransferase (DNMT) and histone deacetyltransferases (HDACs) have already been approved by the US Food and Drug Administration (FDA) [4, 6]. Until recently, there was no small molecule capable of targeting the reader molecule. However, the first generation of bromodomain and extra-terminal domain (BET) protein inhibitors, JQ1 and I-BET, have been developed and their feasibility in inhibiting reader molecules by interfering with protein-protein interaction has been established [7, 8].

The BET family is comprised of BRD2, BRD3, BRD4 and the testis specific BRDT, which recognize lysine acetylation and serve as modification effectors by recruiting additional chromatin modifiers and remodeling enzymes [9, 10]. Among various epigenetic modifications, acetylation of lysine residues is the most abundant modification of cells and a major histone modification involved in chromatin structure [10]. BET family member BRD4 is a RNA polymerase II (Pol II) elongation complex with positive transcription elongation factor complex b (P-TEFb) [11, 12]. Although BRD4 has kinase activity on Pol II [13], generally it couples the acetylation state and plays role for rapid transcription induction, such as mitotic exit and the inflammation process [8, 14]. The BET family regulates diverse genes involved in cellular activities suggesting that it conducts as epigenetic signaling molecule [9, 15]. Although there have been concerns over the non-specific effects of BET protein inhibition, following studies have evidenced that its pharmacological inhibition is relatively specific and beneficial in a variety of diseases [15–19].

Human liver cancer includes diverse, biologically distinct hepatic neoplasms [20] and hepatocellular carcinoma (HCC) is the most common and very aggressive liver cancer. A variety of genetic events have been relevant to the development of HCC including genome instability, suppression of tumor suppressors and overexpression of oncogenes [20, 21]. Along with genetic aspects, epigenetic disruptions including changes in DNA methylation, microRNA expression have also been studied in liver cancer, and mutations or abnormal expression of epigenetic regulatory genes have been recognized [22–24]. Despite these efforts, the underlying mechanism responsible for liver carcinogenesis is largely unknown and therapeutic options remain limited.

In this study, we investigated whether epigenetic reader BRD4 inhibition has therapeutic efficacy in liver cancer. We found that BRD4 is relatively overexpressed in liver cancer and its inhibition induces anti-tumorigenic effects via E2F2-cell cycle regulation circuit not through the MYC, suggesting a feasible therapeutic approach for cancer driven by E2F2-cell cycle regulation by targeting the epigenetic reader BRD4. Furthermore, the results provide innovative insights that targeting epigenome components will be a good therapeutic strategy.

## RESULTS

### Epigenetic reader brd4 is overexpressed in three large hcc cohorts and brd4 inhibition by small molecule jq1 induces anti-tumorigenic effects in liver cancer cells

**Figure 1:**
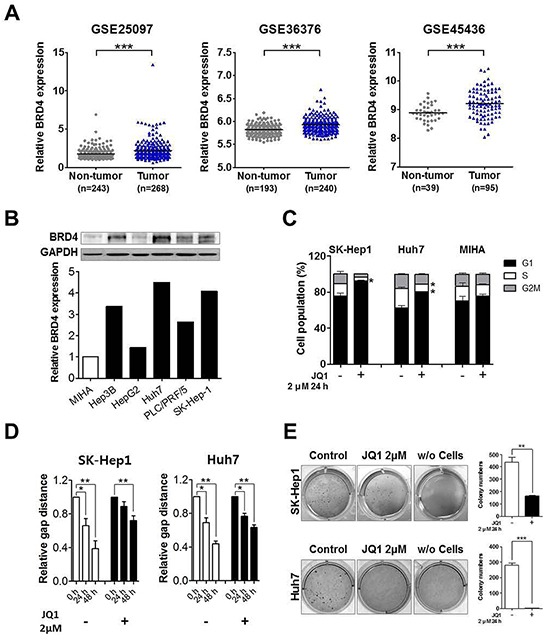
Over-expressed BRD4 inhibition induces anti-tumorigenic effects in liver cancer **A.** GEO data sets of GSE25079, GSE36376 and GSE45436 showed that BRD4 expression was significantly overexpressed in HCC (mean ± S.D., ***p<0.001, versus non-tumor). **B.** Endogenous BRD4 protein expression level was determined by Western blot in liver cancer cell lines and immortalized non-tumorigenic human hepatocyte cell line MIHA. GAPDH is indicated as a loading control and densitometry was used to quantify western blot data **C.** SK-Hep1, Huh7 and MIHA cells were treated with 2 μM JQ1 for 24 h and cell number of each cell cycle were determined by flow cytometry. **D.** SK-Hep1 and Huh7 cells were treated with 2 μM JQ1 for 24 h and then subjected to wounding. Wound healing capacity was determined for 48 h and the data were presented as the mean ± S.E.M. (*p<0.05, **p<0.01, ***p<0.001 vs. control). **E.** Soft agar colony formation was performed with and without JQ1 treatment using SK-Hep1 and Huh7 cells and the colony number is illustrated by bar graph.

To determine whether BRD4 can be a therapeutic target in liver cancer, we first examined the expression of BRD4 in three large publically available cohorts of human HCC patients from the National Center for Biotechnology Information (NCBI) Gene Expression Omnibus (GEO) data base (GSE25097, GSE36376 and GSE45436), since intuitively overexpressed expression is more likely (but not always) functionally relevant. The epigenetic reader BRD4 is significantly overexpressed in tumor tissues than normal in three cohorts (Figure 1A). Next, BRD4 expression was examined by Western blot analysis in five liver cancer cell lines (Hep3B, HepG2, Huh7, PLC/PFR/5 and SK-Hep1) along with the immortalized non-tumorigenic human hepatocyte cell line MIHA. The human liver cancer cell lines exhibited relatively high BRD4 expression, with the exception of HepG2 hepatoblastoma cell line (Figure 1B), indicating that targeting BRD4 could be effective for liver cancer therapy.

JQ1 is a potent inhibitor of BRD4. It was used to assess the sensitivity of liver cancer using SK-Hep1 liver cancer cells. Minor change in cell morphology and cell cycle arrest were evident at 2 uM JQ1, a relatively high concentration compared to other cancer cells. The altered cell cycle composition began at 6 h and clear G1 arrest was apparent at 24 h (Supplementary Figure S1A and S1B). To examine the role of BRD4 in cell proliferation, cell viability was determined using a 3-(4,5-dimethylthiazol-2-yl)-2,5-diphenyltetrazolium bromide (MTT) based assay. JQ1 reduced the growth rate of SK-Hep1 and Huh7 cancer cells, but not MIHA non-tumorigenic hepatocyte cells (Supplementary Figure S1C). Consistent with this result, marked cell cycle arrest effect was observed in liver cancer cell lines (Figure 1C) but not in MIHA. To further study the anti-tumorigenic effect of JQ1, scratch wound healing and soft agar colony formation experiments were done. JQ1 efficiently reduced wound healing capacity and soft agar colony formation in SK-Hep1 and Huh7 cells (Figures 1D, 1E and Supplementary Figure S1D). The collective results indicate that BRD4 could be a therapeutic target in liver cancer and its inhibition by small molecule has anti-tumor efficacy in liver cancer cell lines.

### Brd4 inhibition reverses liver cancer related gene expression signature, but does not involve myc

**Figure 2:**
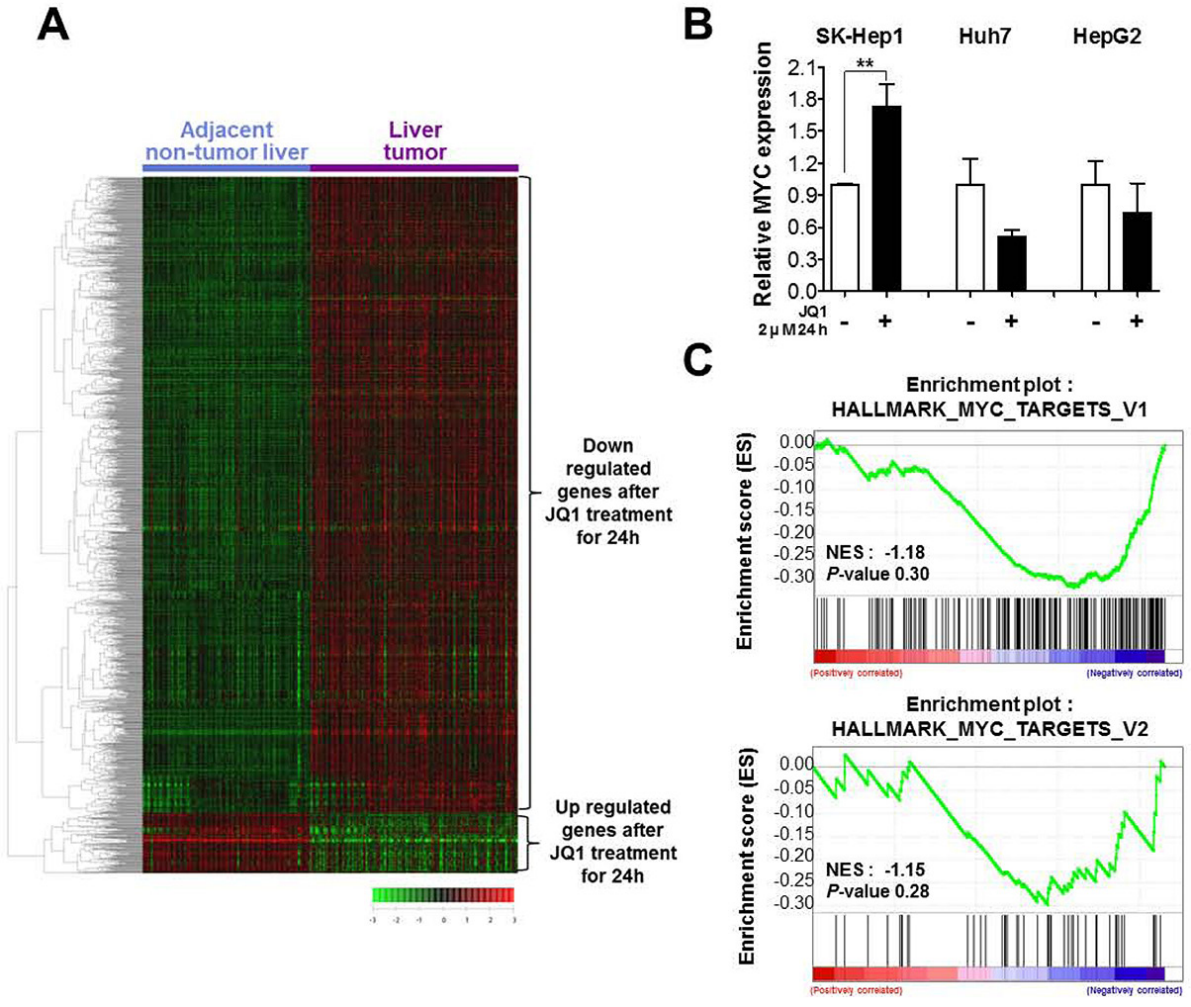
BRD4 inhibition inverts liver cancer related gene expression signature but it is not through suppression of MYC **A.** The microarray data were obtained from the GEO database (GSE36376). Genes (n=1,570) deregulated by 2 μM JQ1 treatment for 24 h in SK-Hep1 cells were selected for cluster analysis and analyzed with GSE36376 compared liver tumor tissue with adjacent non-tumor tissue. **B.** MYC mRNA levels were determined by real-time PCR after treatment of 2 μM JQ1 for 24 h. We performed three independent experiments but there was no significant level change of MYC in Huh7 and HepG2. **C.** GSEA of two Myc-dependent gene sets was performed with JQ1 targets. NES indicates normalized enrichment score.

To obtain a comprehensive transcription landscape after BRD4 inhibition and its clinical significance in liver cancer, we separated JQ1 up- and down-regulated genes and investigated their expression pattern in a large cohort of patients. Down-regulated genes (1,435 gene elements, fold change >1.5) after JQ1 treatment were prominent in liver tumors compared to adjacent non-tumor liver cells, while up-regulated genes (135 gene elements, fold change >1.5) are underexpressed in liver tumors (Figure 2A), suggesting that BRD4 inhibition could reverse liver cancer related gene expression signature.

Most (but not all) studies have shown that anti-tumorigenic effect of BRD4 inhibition is through the antagonization of MYC, which is the most well-known oncogene in several cancers. [15, 19, 25, 26] We examined whether anti-tumorigenic effect of JQ1 is through MYC, firstly we checked MYC expression after treatment of JQ1 in SK-Hep1, Huh7 and HepG2 liver cancer cells. MYC expression level is not significantly changed even a little bit increase in SK-Hep1 (Figure 2B). Next, we performed Gene Set Enrichment Analysis (GSEA) to access the functional relevance of MYC targets and JQ1 responsive genes using two Hallmark of MYC target gene sets. BRD4 inhibition did not significantly affect MYC targets (NES=−1.18, *p*=0.30; NES=−1.15, *p*=0.28, Figure 2C), further confirming that BRD4 inhibition does not induce MYC suppression nor its downstream in liver cancer cell line.

### Jq1 responsive genes can be divided into four groups depending on response time and expression pattern, involving distinct biological pathways

**Figure 3:**
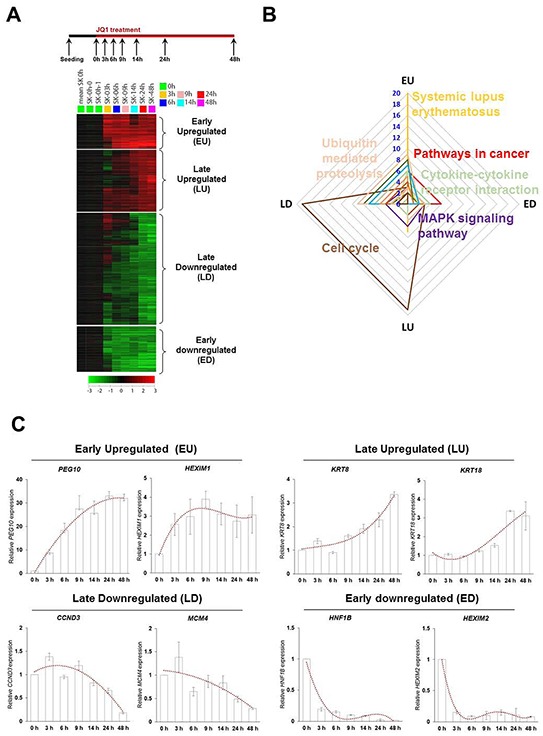
Serial gene expression microarray categories JQ1 responded genes into four groups depend on response time and expression pattern **A.** Differential gene expression profiling and the identification of large-scale molecular changes in JQ1-treated SK-Hep1 cells. These genes are grouped as EU, LU, LD and ED. **B.** Radar graph representing the prevalence of biological pathway relevant to four regulated categories by JQ1 treatment. **C.** The expression pattern of two genes of each group was determined by real time RT-PCR for confirming microarray data.

To delineate the molecular gene expression kinetics of BRD4 inhibition by which executes the effects, we performed serial gene expression analyses from 0 to 48 h after treating SK-Hep1 with JQ1. JQ1 treatment did not prompt non-specific transcriptional silencing. Instead, four groups could be categorized depending on early (3 h to 9h) and late (24 h to 48 h) response timing and up- or down-regulated expression: Early Upregulated (EU, 118 gene elements), Late Upregulated (LU, 210 gene elements), Late Downregulated (LD, 155 gene elements) and Early Downregulated (ED, 391 gene elements) (Figure 3A). KEGG pathway analysis was conducted with the differentially expressed genes in each group. The relevance of group and biological pathways depicted using a radar graph (Figure 3B) illustrated the interaction and correlation of pathways between the EU, ED, LU and LD categories. Blue numbers denote the number of genes in each pathway. The EU group was highly enriched with systemic lupus erythematosus, an autoimmune disease [27] (Figure 3B, yellow line). At early time points, the cells might sense JQ1 as a viral infection or toxic reagents and activate their immune system to protect themselves similar to the recently demonstrated mechanism of DNA methylation inhibition [28, 29]. This remains to be conclusively established. The LU and LD groups showed high enrichment in cell cycle related pathway, which is critical to cancer proliferation (Figure 3B, brown line). ED group showed high enrichment in several cancer related pathways (Figure 3B, red line), such as cell cycle, focal adhesion and repair which may connotes that liver cancer cells may be threatened their identity. To validate microarrays and to confirm transcriptional levels of differentially expressed genes of four individual group by JQ1 treatment, we selected significantly changed genes from microarray data of JQ1 and performed real time RT-PCR analysis (Figure 3C). The expression of two select genes in each group, EU (PEG10, HEXIM1), LU (KRT8, KRT18), LD (CCND3, MCM4) and ED (HNF1B, HEXIM2), were changed in expected time and pattern.

### E2f2 is the direct target of brd4 inhibition in liver cancer cells and jq1 down-regulate e2f2 directly by suppressing brd4 binding at its proximal promoter

**Figure 4:**
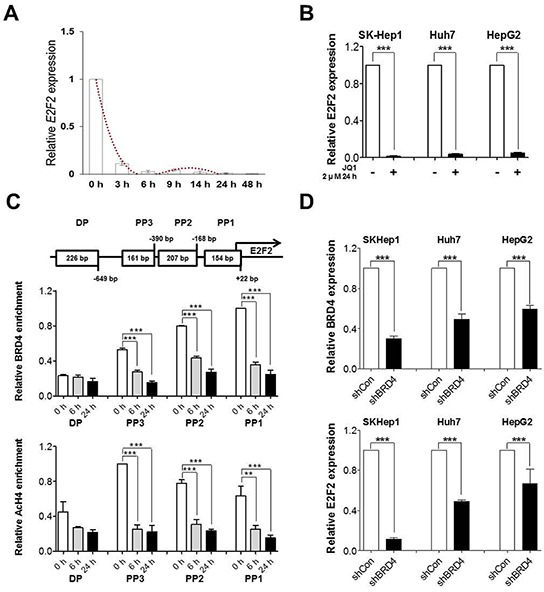
JQ1 downregulates E2F2 directly via suppression of BRD4 binding at proximal promoter and further confirmed by loss of BRD4 **A.** and **B.** E2F2 mRNA levels were determined by real-time PCR after treatment of JQ1 in SK-Hep1, Huh7 and HepG2. **C.** Schematic diagram of the E2F2 promoter upto upstream 1 kb. Chromatin was immunoprecipitated with anti-BRD4 and anti-AcH4 antibodies. BRD4 binding and enrichment of acetylated histone4 at the E2F2 regulatory regions was analyzed by real-time PCR. **D.** E2F2 mRNA levels were determined by real-time PCR after loss of BRD4 expression in SK-Hep1, Huh7 and HepG2. Data are presented as the mean ± S.E.M. (*p<0.05**p<0.01, ***p<0.001 vs. control).

To identify the key target of BRD4 inhibition, we focused on the ED group, which presumably has direct targets of BRD4 inhibition, since BRD4 recognizes acetylated lysine residues relevant to transcription activation, and since previous studies showed that BRD4 inhibition by JQ1 occurs early [7, 9, 15]. PANTHER protein class analysis with ED revealed great enrichment of nucleic binding protein and transcription factor (TF) in the ED group (Supplementary Figure S2A and S2B). E2F2, which belongs to the crucial cell cycle regulator E2F family [30], was the most down-regulated gene among the ED group of genes (Supplementary Figure S2C). Real time RT-PCR confirmed E2F2 suppression by JQ1 in SK-Hep1 cells (Figure 4A). Down-regulation of E2F2 was evident at a very early time point (3 h) and its suppression was also detected in Huh7 and HepG2 liver cancer cells (Figure 4B). MYC expression was not down-regulated until 24 h was detected 48 h, confirming that MYC is not direct target of BRD4 inhibition, but that suppression later in time may be a secondary effect (Supplementary Figure S3A).

To examine whether downregulation of E2F2 by JQ1 is a result of direct inhibition by BRD4 binding, we performed chromatin immunoprecipitation (ChIP) using BRD4 antibody. BRD4 was highly enriched at the proximal promoter of E2F2 in SK-Hep1 cells and its binding decreased early upon treatment (6 h) with JQ1, which was correlated with E2F2 transcription in a time-dependent manner (Figure 4C). ChIP was performed using Acetylated histone H4 (AcH4) antibody, a well-known active promoter marker, to depict the epigenetic landscape of E2F2 promoter after treatment of JQ1. E2F2 promoter regions were hypo-acetylated with BRD4 inhibition, which correlated with E2F2 expression state (Figure 4C).To further confirm whether BRD4 is required for the overexpression of E2F2, we performed a loss of function study by generating stable BRD4 knockdown cells using shRNA in SK-Hep1, Huh7 and HepG2 liver cancer cells. As shown in Figure 4D, reduced expression of E2F2 was evident after BRD4 knockdown. Furthermore, loss of BRD4 mimicked the anti-tumorigenic effect of JQ1 including reduced wound healing capacity and soft agar colony formation (Supplementary Figure S3B). Taken together, the results demonstrate that E2F2 is a direct target of the epigenetic reader BRD4 in liver cancer cells.

### E2f2 is the first-line target of brd4 inhibition and the genes are enriched cell cycle regulation

**Figure 5:**
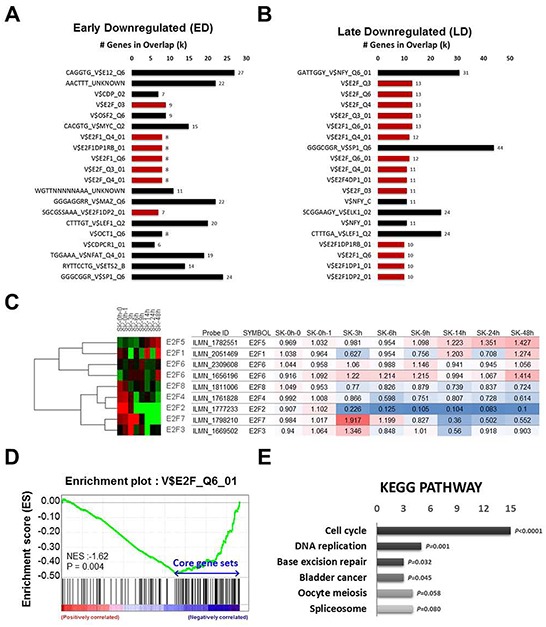
E2F2 is the first line target of BRD4 inhibition **A.** and **B.** Upstream motif analysis using MSigDB were performed in early and late downregulated groups. E2F binding motif was colors with red. **C.** Relative gene expression change of E2F family after treatment of JQ1 was presented by heatmap and colored table. **D.** and **E.** GSEA and KEGG pathway analysis were performed with E2F motif and JQ1 targets.

To determine if E2F2 suppression could explain the gene expression changes by BRD4 inhibition, we performed upstream motif analysis using the Molecular Signatures Database (MSigDB). Surprisingly, E2F transcription factor binding motif (red color) was frequently detected (7 and 14 of top 20, respectively) in the ED and LD groups (Figure 5A and 5B), but not in the upregulated groups (Supplementary Figure S4A and S4B). Notably, only E2F2 was significantly down-regulated by JQ1 among the E2F family TFs in SK-Hep1 cells (Figure 5C), suggesting that BRD4 inhibition is specific to E2F2 in the E2F family.

To test the hypothesis that BRD4 inhibition will specifically abrogate E2F2 dependent transcription, we performed GSEA and confirmed that genes down-regulated by BRD4 inhibition were significantly enriched for the E2F DNA binding motif (NES=−1.62, *p*<0.004), presumably E2F2 in this case (Figure 5D). Further, to examine the influences on biological networks regulated by E2F2 and BRD4, KEGG pathway analysis with 107 core genes demonstrated that BRD4 and E2F2 common targets are enriched in cell cycle and DNA replication (Figure 5E). To gain further biological function insights for E2F2, we analyzed the Molecular Concept Map and identified an enrichment network linking E2F2 signatures with cell cycle regulation (Supplementary Figure S4C). Altogether, these data demonstrate that the anti-tumorigenic effect of JQ1 is through the down-regulation of E2F2 and sub-sequential suppression of its downstream cell cycle regulation circuit.

### Brd4-e2f2-cell cycle regulation circuit is highly activated in human HCC tissues and high e2f2 expression is associated with poor prognosis of HCC patients

**Figure 6:**
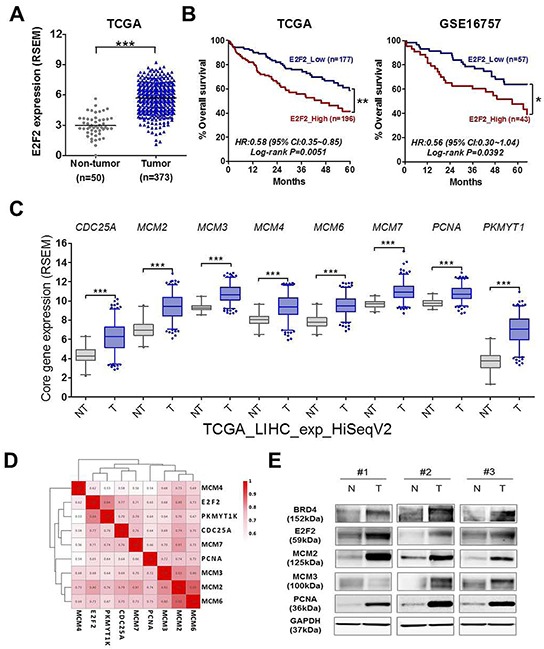
BRD4-E2F2-cell cycle regulation circuit is highly activated in human HCC tissues and high E2F2 expression is associated with poor prognosis of HCC patients **A.** The relative E2F2 gene expression levels in non-cancerous tissue (Non-tumor) and HCC patients’ tissue (Tumor) was illustrated by scatter blot using TCGA data set. The median expression level of each group was indicated by horizontal lines. **B.** Overall survival dependent on E2F2 expression was shown by Kaplan-Meier survival curves. P-values were obtained with the log-rank test. **C.** The relative level of represented core genes of BRD4 inhibition and E2F target including CDC25A, MCM2, MCM3, MCM4, MCM6 MCM7, PNCA and PKMYT1 were illustrated with box plot using TCGA dataset. **D.** The correlation analysis was performed between E2F2 and 8 core genes expression and represented by correlation heatmap. The numbers indicate R values which calculated based on Pearson correlation coefficient. **E.** BRD4, E2F2, MCM2, MCM3, PCNA and GAPDH protein levels were analyzed by western blot in three human HCC tissues paired with histologically normal liver tissue.

To investigate the clinical significance of E2F2 in liver cancer, we analyzed its expression using the NCBI GEO data base (GSE25097, GSE36376 and GSE45436) and TCGA data set. E2F2 was significantly up-regulated in three large cohorts and TCGA data set of patients with HCC (Supplementary Figure S5A and Figure 6A). High expression of E2F2 was significantly associated with poor prognosis of patients with HCC in the TCGA dataset (Kaplan-Meier plot; HR, 0.58; 95% CI, 0.35 to 0.85; *p=* 0.0051) and GSE16757 cohort (Kaplan-Meier plot; HR, 0.56; 95% CI, 0.30 to 1.04; *p* = 0.0392) (Figure 6B). Consistently, 8 core genes of BRD4 inhibition and E2F target such as Cell Division Cycle 25A (CDC25A), Minichromosome Maintenance Complex Components (MCMs) and PNCA are overexpressed in tumors than normal tissues (Figure 6C and Supplementary Figure S5B) and there was a positive correlation among the 8 core genes and E2F2 (Figure 6D). Of note, Western blot analysis of HCC subset tissues showed that BRD4-E2F2-cell cycle regulation axis is overexpressed (Figure 6E), highlighting critical role of E2F2 in liver cancer. Taken together, these data suggest that E2F2 identified by BRD4 inhibition is a novel target for control of cell cycle in liver cancer.

## DISCUSSION

We investigated the activity of BET protein inhibitor in liver cancer. E2F2-cell cycle regulation circuit was revealed as a major target of BRD4 inhibition. BRD4 was overexpressed in liver cancer cell lines and liver tumor tissue, compared to than normal in three large cohorts. BRD4 inhibition by JQ1 induced anti-tumorigenic effects including cell cycle arrest, reduced wound healing capacity and soft agar colony formation in liver cancer cell lines. BRD4 inhibition by JQ1 selectively repressed transcriptional networks induced by E2F2 not through MYC and inverts liver cancer related gene expression signature. Serial gene expression analyses from 0 to 48 h after treating SK-Hep1 with JQ1 categorized JQ1 responsive genes into four subgroups dependent on the response time and expression pattern. Each group had distinct biological pathways. We focused on the ED group, which more likely has direct targets of BRD4 inhibition; the group was greatly enriched in cancer related categories. Protein classification analysis revealed high enrichment of TF in the ED group, especially with E2F2. Anti-BRD ChIP assay and loss of function experiments for BRD4 demonstrated that E2F2 as the direct target of BRD4. Notably, both of ED and LD groups frequently harbored the E2F binding motif in their upstream sequences, suggesting that E2F2 is the key molecule for efficacy of BRD4 inhibition especially for the suppressed genes. GSEA also showed a clear negative correlation with JQ1 responded genes with E2F targets and the core gene set was enriched in cell cycle. Indeed, E2F2 was up-regulated in three large cohorts of HCC patients and several core genes were overexpressed in HCC tissue. Over-expression of the BRD4-E2F2-cell cycle regulation axis was evident in tumor tissue from the HCC patients. Lastly, over-expression of E2F2 was significantly associated with poor prognosis of patients with HCC, demonstrating that BRD4-E2F2-cell cycle regulation circuit is a novel target in liver cancer. Taken together, we demonstrate that targeting epigenetic reader by small molecule will be a good therapeutic strategy in liver cancer with underlying E2F2 down-regulation.

MYC is the best characterized proto-oncogene. MYC is the primary target of BET family inhibition in several cancers [7, 15, 19], and the underlying mechanism is proposed to involve JQ1 inhibiting MYC by disrupting super enhancers, which are defined as large clusters of enhancers that determine cellular identity [31]. However, it is not clear whether MYC has a super enhancer in different cellular contexts and MYC is always the main target of BET protein inhibition. Recent reports suggested that the efficacy of BET inhibitors is dependent on other molecules, such as FOSL1, HEXIM1, TWIST and RelA [18, 32–34]. We thought that BET inhibitors may function differently in a cell context dependent manner and so it was important to define the main target and underlying mechanism of BET inhibitors in different cellular contexts to ascertain efficacy. Indeed, our data demonstrate that BET inhibitor has anti-tumorigenic effects in liver cancer cell line, but not through MYC.

Using serial expression microarray and loss of function experiments followed integrated mining, we revealed that E2F2 is the first-line target of BRD4 inhibition and a promising therapeutic target in liver cancer. E2F family TFs are known downstream effectors of the retinoblastoma (RB) protein and crucial for cell cycle progression, differentiation and apoptosis [30, 35, 36]. There are at least 8 different E2F TFs and they can act as activator or repressor dependent on the cell context [30, 37]. E2F2 is up-regulated in breast cancer and glioblastoma [38–40]. Interestingly, loss of E2F2 in human embryonic stem cells inhibits tumorigenicity specifically without affecting pluripotency [41], highlighting E2F2's distinct function compared to other E2F families. It would be worth determining whether E2F2 is a putative cancer stem cell marker and whether BET protein inhibition could be therapeutic.

Notably, tumor suppressive activities of E2F2 have been reported in MYC triggered tumorigenesis [42–44]. However, E2F2's oncogenic activities also have been demonstrated with MYC dependency [45, 46]. Therefore, E2F2 seems to have a different role dependent on the cellular context and environment. In our system, significant overexpression of E2F2 in the liver cancer cohorts was evident, and TCGA data set and downregulation of E2F2 after JQ treatment was more significant and general event than MYC downregulation, demonstrating that E2F2 more likely acts as a key oncogene in liver cancer and anti-tumorigenic effect of JQ1 is probably mediated by downregulation of oncogenic activity of E2F2. A better understanding of the relationship between E2F2 and MYC in various cancer contexts remains for future study.

While preparing our manuscript, two papers were published reporting that BRD4 is responsible for tumor growth in HCC [47, 48]. However, the two studies did not feature kinetic data. Similar to us, Zhang et al.[47] demonstrated that BRD4 over-expression is correlated with poor prognosis in HCC patients. However, we could not observe any effect on epithelial mesenchymal transition (EMT) by BRD4 inhibition in SK-Hep1 (Supplementary Figure S6). SK-Hep1 cells highly express mesenchymal molecules including TWIST1, SNAI1, SNAI2 and VIM, but their expression was not changed significantly by JQ1 (Supplementary Figure S6A). We further analyzed BRD4-E2F2 level in HCC patients with vascular invasion and 11 HCC patients with portal vein tumor thrombus (PVTT) which is one of the most serious complications of HCC with metastasis. There was no significant relevance between BRD4-E2F2 axis and EMT process in liver cancer (Supplementary Figure S6B and S6C). Overexpression of BRD4 may function for initiating EMT but BRD4 inhibition in already established mesenchymal states may not be enough to convert the state. Li et al.[48] showed that JQ1 induced cell cycle arrest by repressing MYC expression, which we did not find. The detailed molecular mechanism of these issues requires further investigation.

Extensive cancer genome studies have been done since genome instability and accumulation of genetic mutations have been thought as major underlying mechanisms during tumorigenesis [49]. In liver cancer studies, chromosomal aberrations and mutations of tumor suppressors or oncogene have been reported and discussed for decades. However, targetable molecules that are growth factor receptor or protein kinases mutation rate is not high in HCC, emphasizing the need for finding other targets for the treatment of liver cancer. Of note, recent exome sequencing studies have revealed several new cancer genes belonging to epigenome components, further demonstrating that genetic and epigenetic regulations are more intertwined than has been expected [5, 50, 51]. Besides genetic mutation screening, epigenetic characterization including expression level of epigenetic components may provide valuable information for understanding tumorigenesis and further development of new drugs for cancer.

Taken together, we found that over-expression of epigenetic reader BRD4 in liver cancer and for the first time demonstrate that the BET protein inhibitor suppresses E2F2 itself and its downstream cell cycle regulation circuit in liver cancer. Our study suggests that a better understanding of epigenetic components will provide new targets and facilitate the development of novel strategies for treating intractable cancer such as HCC.

## MATERIALS AND METHODS

### Tissue sample

Total theree HCC patient tissues with their corresponding normal tissues were obtained from the Liver Cancer Specimen Bank of the National Research Resource Bank Program of the Korea Science. Written informed consent was obtained from each subject according to the Declaration of Helsinki, and the study was approved by the Institutional Review of Board (IRB) of the College of Medicine (Songeui Campus) of the Catholic University of Korea (IRB approval number: MC12SNMI0184).

### Cell culture

The human HCC cell lines Hep3B, HepG2, Huh7, PLC/PRF/5, SK-Hep-1 were obtained from KCLB (Korean Cell Line Bank, Seoul, South Korea). The immortalized non-tumorigenic human hepatocyte cell line MIHA was kindly provided by Dr. Roy-Chowdhury (Albert Einstein College of Medicine, NY, USA). Each cell line was maintained in RPMI-1640, DMEM (Lonza, Walkersville, MD) or EMEM (ATCC, Manassas, VA) supplemented with 10% fetal bovine serum (FBS; Sigma-Aldrich, St. Louis, MO) and 100 units/mL of penicillin–streptomycin (Invitrogen, Carlsbad, CA). All cells were cultured at 37°C in a humidified incubator with 5% CO^2^.

### shRNA infection

shBRD4 constructs were purchased from Sigma-Aldrich. For lentivirus production, MISSION lentiviral packaging mix was used. Infected derivative cells stably expressing shRNA were selected in the presence of 1.25 μg/mL puromycin.

### RNA extraction and reverse transcription PCR

Total RNA was extracted using TRIzol reagent, digested with DNase I and reverse transcribed using a High Capacity cDNA Reverse Transcription Kit (Applied Biosystems, Foster City, CA). Amplification of cDNA was performed on a LightCycler® 480II (Roche, Basel, Switzerland) using the LightCycler® 480 SYBR Green I Master (Roche), according to the recommended conditions. cDNAs were amplified using the following gene-specific primers and the primer sequences are available upon the request.

### Western blot analysis

Cells were lysed with RIPA buffer and sonicated briefly. Cell lysates were boiled in Laemmli sample buffer, and 30 μg of each protein was subjected to SDS-PAGE. The protein concentration was measured by Bradford protein assay. Antibodies against BRD4 (Cell Signaling Technology, Danvers, MA; cat#13440), E2F2 (AbFrontier, Seoul, Korea; YF-PA11461), MCM2 (Cell Signaling Technology; cat#3619), MCM3 (Cell Signaling Technology; cat#4003), PCNA (Cell Signaling Technology; cat#13110), glyceraldehyde-3-phosphate dehydrogenase GAPDH; Santa Cruz Biotechnology, Santa Cruz, CA; sc-32233) were purchased from the indicated companies.

### Chromatin immunoprecipitation assay (ChIP)

ChIP assays were performed according to instructions from Upstate Biotechnology (Lake Placid, NY). For each assay, 50 μg DNA, sheared by a sonication (the DNA fragment size was 200 to 500 bp), was pre-cleared with protein A magnetic beads (Upstate Biotechnology; cat. #16-661) and then 40 μg DNA was precipitated by BRD4 (Cell Signaling Technology; cat#13440) or by Acetylated histone H4 (Millipore, Billerica, MA; cat#06-866). After immunoprecipitation (IP), recovered chromatin fragments were subjected to real-time PCR. IgG control experiments were performed for all ChIPs and incorporated into the IP/Input (1%) by presenting the results as (IP- IgG)/(Input-IgG). ChIP primer sequences are available upon the request.

### Gene expression microarray

Total RNA was amplified and purified using TargetAmp-Nano Labeling Kit for Illumina Expression BeadChip (EPICENTRE, Madison, WI) to yield biotinylated cRNA according to the manufacturer's instructions. Detection of array signal was carried out using Amersham fluorolink streptavidin-Cy3 (GE Healthcare Bio-Sciences, Little Chalfont, UK) according to the bead array manual. Arrays were scanned with a bead array reader confocal scanner according to the manufacturer's instructions (Illumina, San Diego, CA). The quality of hybridization and overall chip performance were monitored manually by visual inspection of both internal quality control checks and the raw scanned data. Raw data were extracted using the software provided by the manufacturer (Illumina GenomeStudio v2011.1, Gene Expression Module v1.9.0). Array probes were logarithm-transformed and normalized by the quantile method.

### Cell cycle analysis

After treatment JQ1 with indicated condition, cells were collected by trypsinization and performed cell cycle assays using the Cycletest Plus DNA Reagent Kit (BD Biosciences, Franklin Lakes, NJ), according to the manufacturer's instructions. The profiles of cells in the cell cycle were analyzed using a FACScan flow cytometer (BD Biosciences).

### Soft agar colony forming assay

The assay was performed in 6-well plates. A bottom layer of agar (0.5%) with enriched DMEM media (final 10% FBS) was poured first. After the bottom agar solidified, MAMB231 cells (1.0 x10^4^) were seeded in top agar (0.3%) with enriched DMEM supplemented with 10% FBS and incubated at 37°C for 21 days. The culture medium was changed one or twice weekly. Colonies were visualized by staining for 1 h with 0.005% crystal violet.

### Wound healing assay

Cells were grown to confluence in 6-well plates and treated with 2 μM JQ1. After overnight starvation in serum-free medium, cell monolayers were scraped with a sterile micropipette tip. Initial gap widths (0 h) and residual gap widths at 24 h and 48 h after wounding were determined from photomicrographs.

### GEO data analysis

To analyze the expression level of BRD4 and other genes in HCC, mRNA expression data sets were obtained from the National Center for Biotechnology Information (NCBI) Gene Expression Omnibus (GEO) database (Accession No. GSE25097, GSE36376, GSE45436, GSE20140 and GSE69164).

### TCGA data analysis

To determine whether reduced E2F2 levels correlated with expression of cell cycle core genes in larger HCC cohort, we obtained RNA-seq-based gene expression data from The Cancer Genome Atlas (TCGA) liver hepatocellular carcinoma project. RNA-seq data were analyzed by first replacing all RSEM values identically equal to zero with the smallest nonzero RSEM value, and then a log2 transformation was applied.

### Gene set enrichment analysis

Gene sets were downloaded from the Broad Instituted's MSigDB (http://software.broadinstitute.org/gsea/msigdb). Gene set permutations were used to determine statistical enrichment of the gene sets using the signal-to-noise ratio of JQ1 target genes versus MYC and E2F targets genes.

### Statistical analyses

Results are expressed as mean ± SEM. Most statistical comparisons were calculated by one-way ANOVA followed by Bonferroni's *post hoc* test using GraphPad Prism. A *p* value < 0.05 was considered significant.

### Data access

Expression profiling data of kinetics after JQ1 treatment in liver cancer cell has been deposited in the Gene Expression Omnibus under accession code: GSE75908.

## SUPPLEMENTARY FIGURES


