# Supplementary material for: Epigenetic reader BRD4 inhibition as a therapeutic strategy to suppress E2F2-cell cycle regulation circuit in liver cancer
Source: Oncotarget. 2016 Apr 12;7(22):32628–40. doi: 10.18632/oncotarget.8701 (PMC5078039; doi:10.18632/oncotarget.8701)
Supplement: Supplementary file 1 [file oncotarget-07-32628-s001.pdf]

Epigenetic reader BRD4 inhibition as a therapeutic strategy to suppress E2F2-cell cycle regulation circuit in liver cancer

SUPPLEMENTARY FIGURES

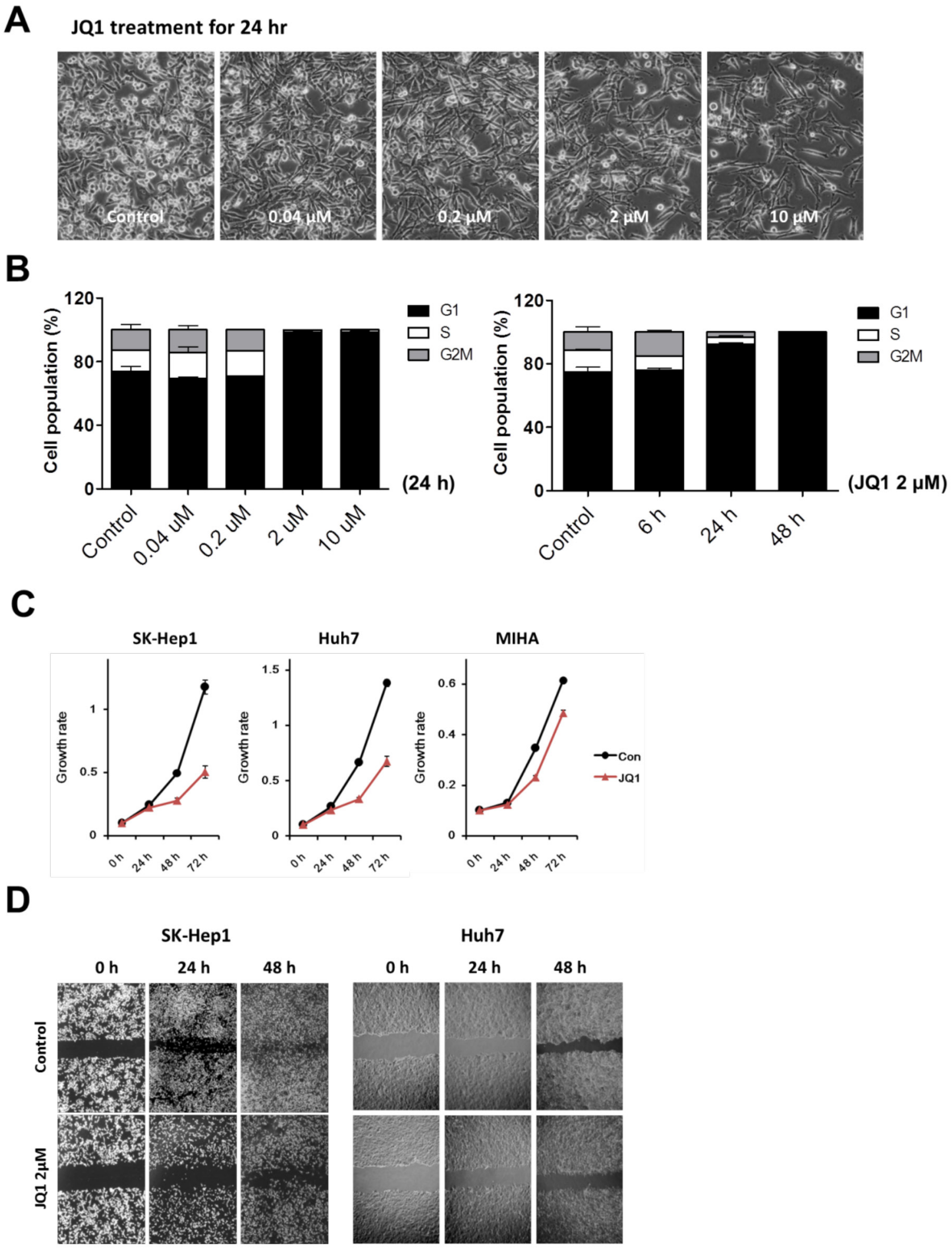

Supplementary Figure S1: JQ1 induces anti-tumorigenic effects on liver cancer cell lines. SK-Hep1 cells were treated with increasing concentrations of JQ1 for 24 h. **A.** Cell morphology at 100X magnification. **B.** DNA content was determined by flow cytometry at the indicated concentration of JQ1 and time points. **C.** SK-Hep1, Huh7 and MIHA cells were treated with 2 µM JQ1 and cell growth was determined by MTT assay. **D.** SK-Hep1 and Huh7 cells were treated with 2 µM JQ1 for 24 h and then subjected to wounding. Wound healing capacity was determined for 48 h.

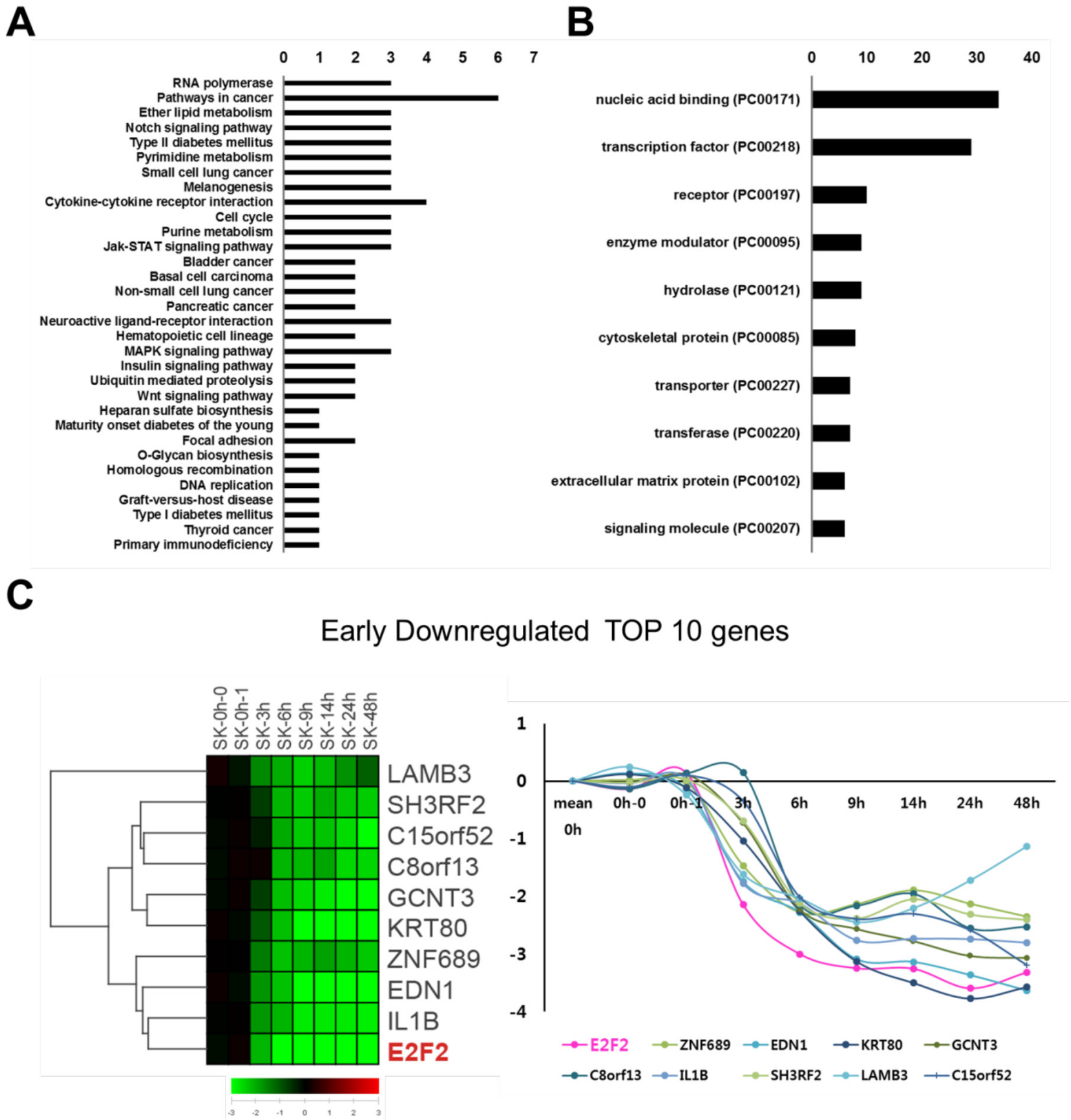

Supplementary Figure S2: Early down regulated genes are major target of JQ1. A and **B.** Determination of characteristic gene expression signatures for JQ1 and functional classification using DAVID. The bar graph shows molecular functional clusters and classification of responsible proteins resulted in genes downregulated at early times with JQ1 treatment. The bar graph is in the order of p-value. **C.** Top 10 downregulated genes at early times with JQ1 treatment were identified and are shown as a heatmap. Fold-changes in the expressions of these 10 genes were plotted at each time point. Fold differences were calculated after subtracting mean values of controls (0 h) from those of samples (3-48 h).

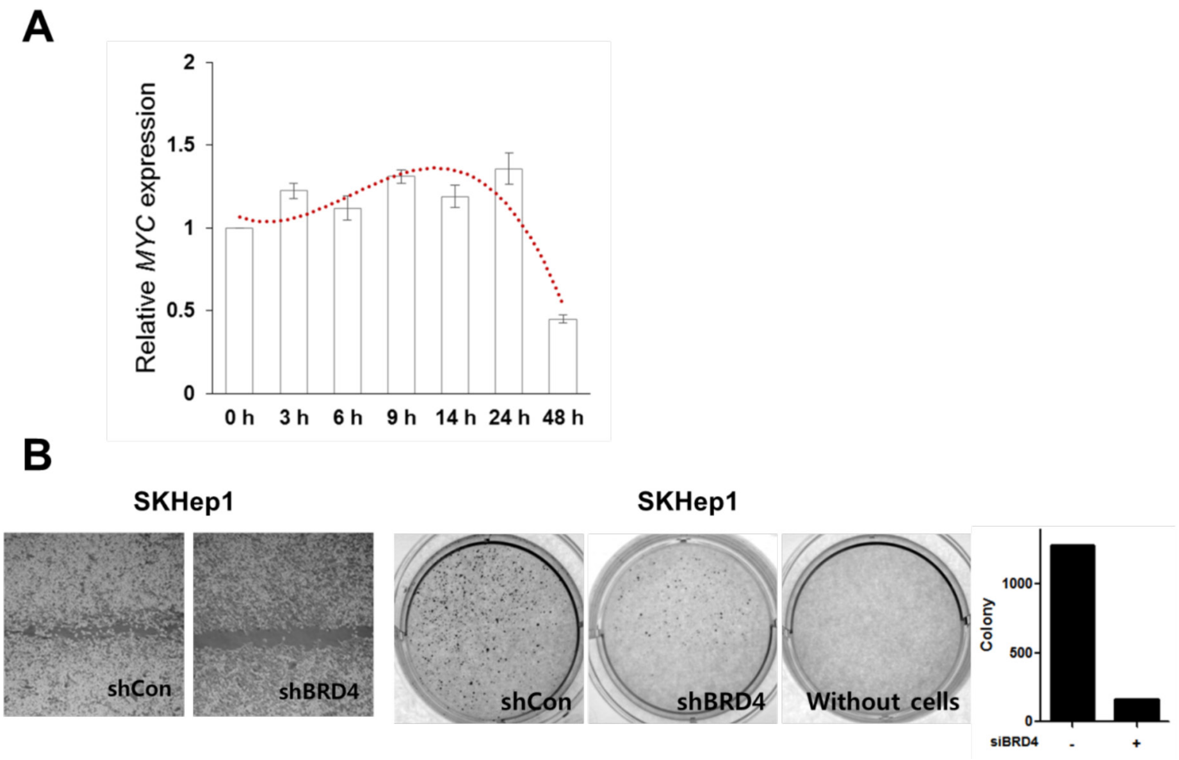

Supplementary Figure S3: MYC is not down-regulated by JQ1 in SK-Hep1. A. MYC mRNA levels were determined by real-time PCR after treatment of JQ1 in SK-Hep1. **B.** Wound healing capacity and soft agar colony formation was performed with SK-Hep-1 after BRD4 knockdown by shRNA. The number of colonies in each plate is depicted in the bar graft.

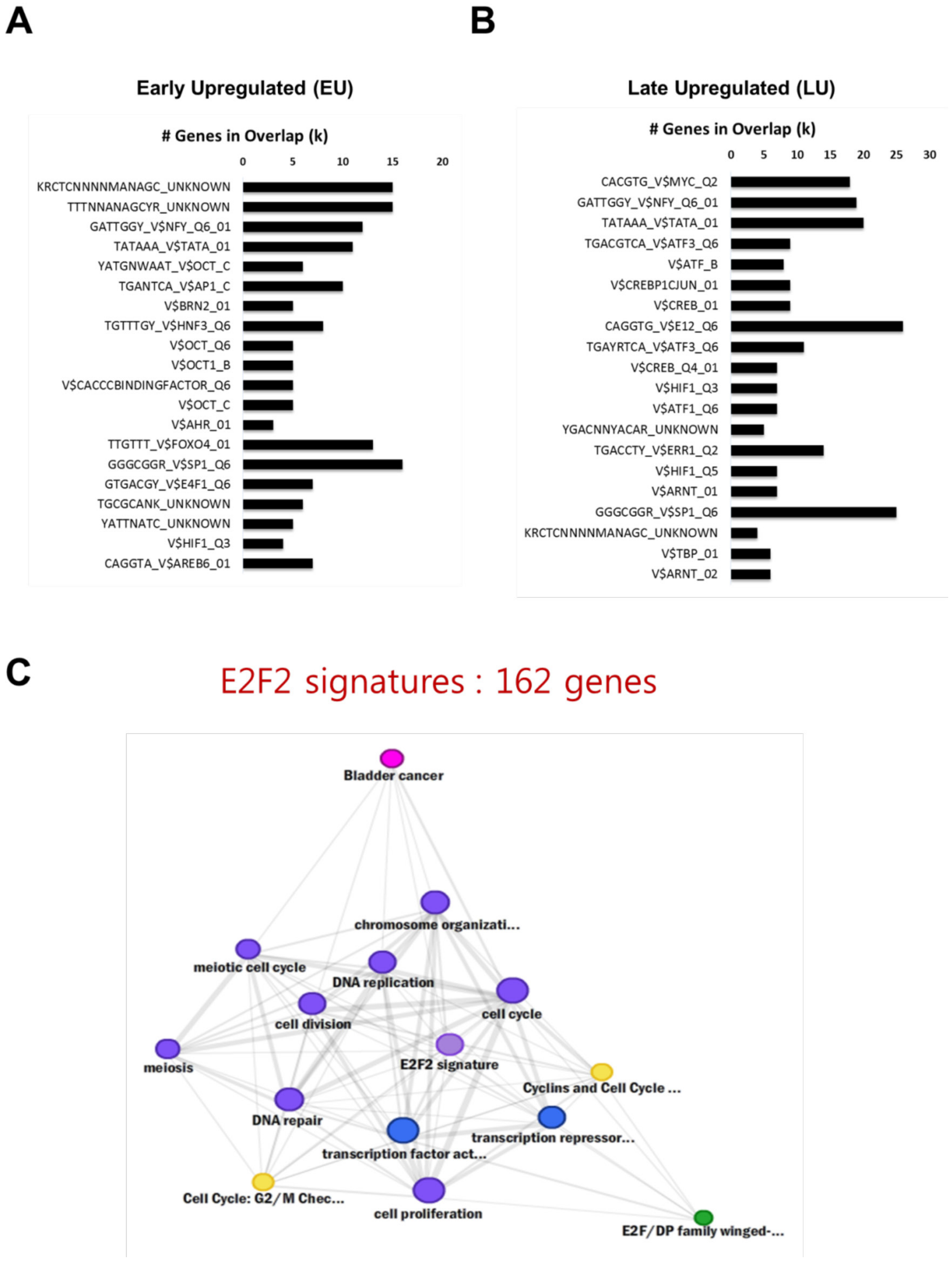

Supplementary Figure S4: Upstream motif analysis of EU and LU gene sets and network analysis of E2F2 signature. A and **B.** Upstream motif analysis using MSigDB were performed with early and late upregulated gene sets. The number of genes overlapping with each motif category are depicted in the bar graft. **C.** An enrichment network linking E2F2 signature with cellular pathways. E2F2-related genes were analyzed using the molecular concept map (ConceptGen). Each node represents one molecular concept or gene set.

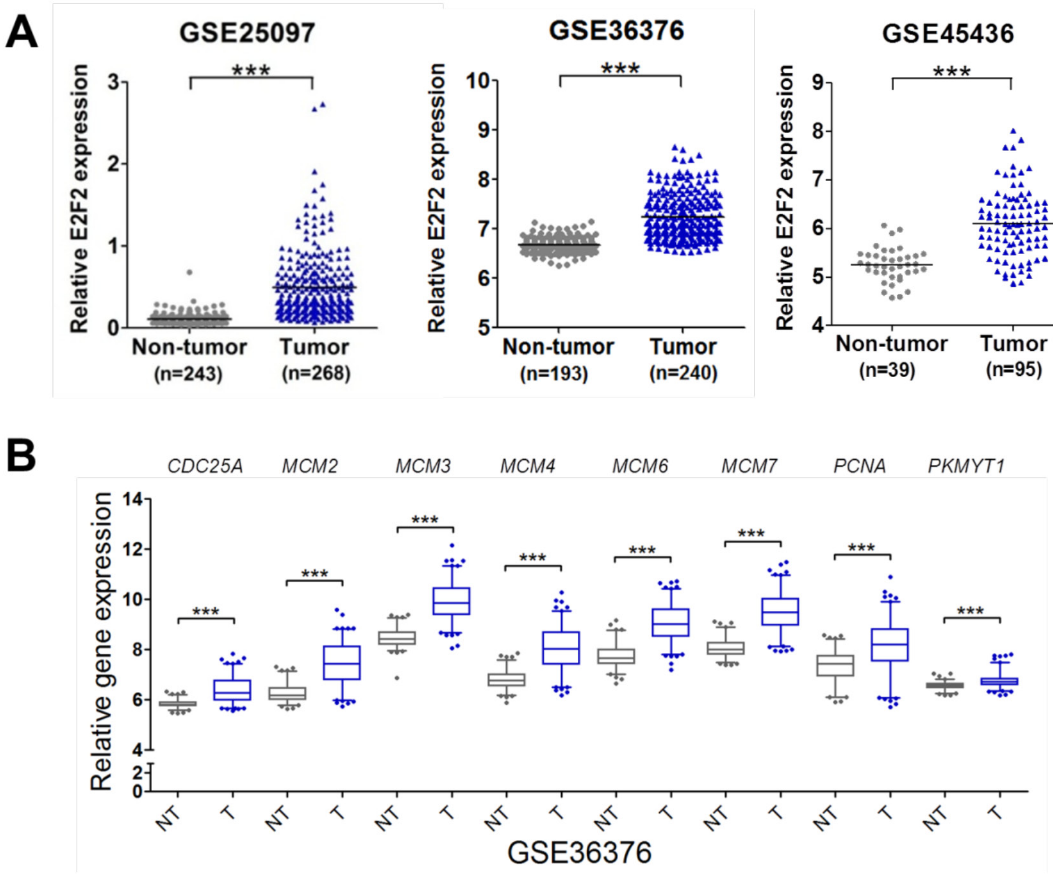

Supplementary Figure S5: BRD4-E2F2-cell cycle regulation circuit is highly activated in human HCC tissues. **A.** The relative E2F2 gene expression levels in non-cancerous tissue (Non-tumor) and HCC patients' tissue (Tumor) was illustrated by scatter blot using three large cohorts GEO data sets (accession numbers GSE25097, GSE36376 and GSE45436). The median expression level of each group is indicated by the horizontal lines. **B.** The relative level of represented core genes of BRD4 inhibition and E2F target including CDC25A, MCM2, MCM3, MCM4, MCM6 MCM7, PNCA and PKMYT1 are illustrated with a box plot using one HCC large cohort (GSE36376).

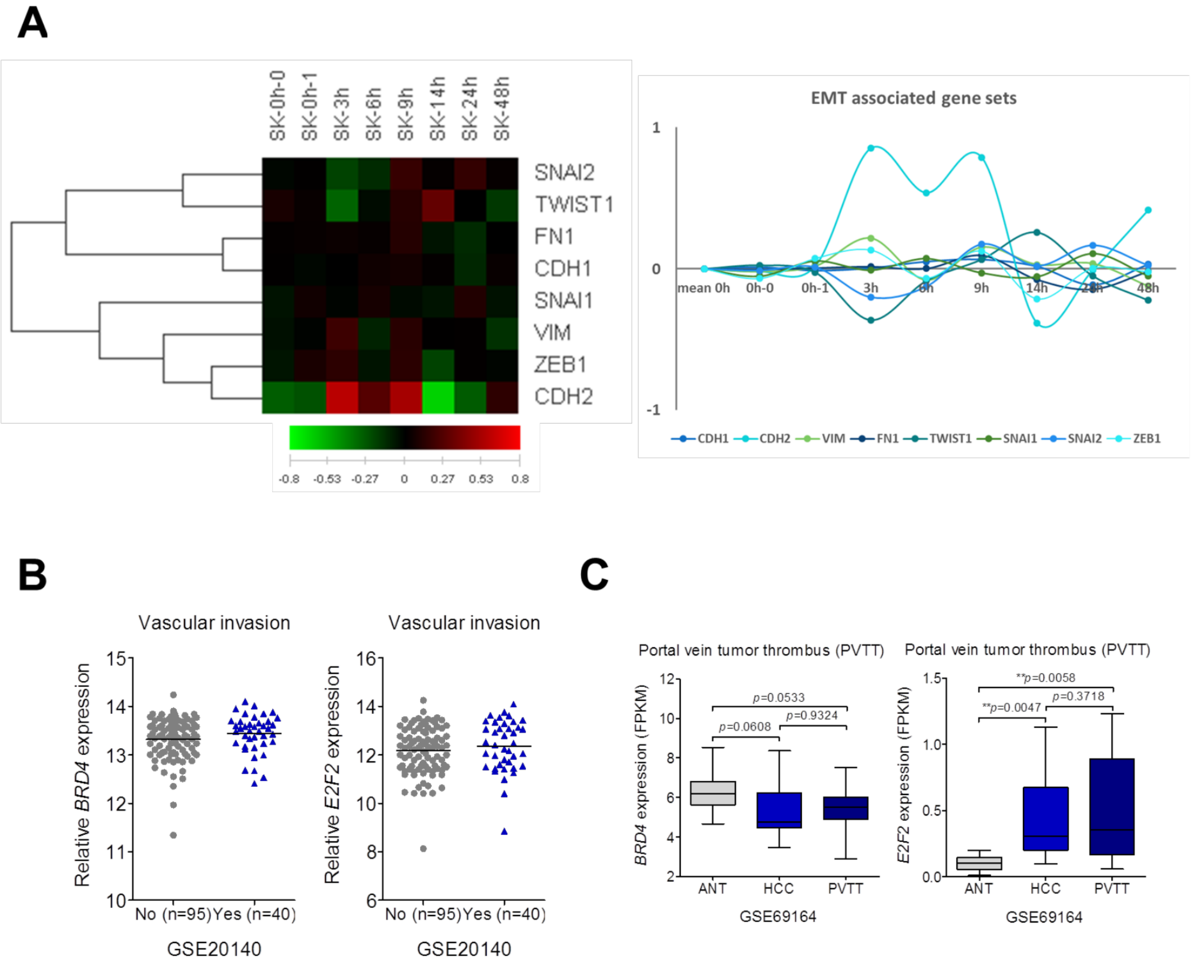

Supplementary Figure S6: JQ1 does not effect on EMT related molecules in SK-Hep1 cells. A. EMT related molecules after JQ1 treatment JQ1 in SK-Hep-1 were identified and shown as heatmap. Fold-changes in the expressions of these 10 EMT molecules were plotted at each time point. Fold differences were calculated after subtracting mean values of controls (0 h) from samples acquired from 3-48 h. **B.** The relative BRD4 and E2F2 expression levels was illustrated by scatter blot in large cohorts associated with vascular invasion in HCC (accession number GSE20140). **C.** The level of BRD4 and E2F2 was illustrated by box plot in large cohort with portal vein tumor thrombus (PVTT) (accession number GSE20140).
